# Chronic Microglial Activation in the GFAP-IL6 Mouse Contributes to Age-Dependent Cerebellar Volume Loss and Impairment in Motor Function

**DOI:** 10.3389/fnins.2019.00303

**Published:** 2019-04-03

**Authors:** Erika Gyengesi, Alejandra Rangel, Faheem Ullah, Huazheng Liang, Garry Niedermayer, Rustam Asgarov, Madhuri Venigalla, Dhanushka Gunawardena, Tim Karl, Gerald Münch

**Affiliations:** ^1^Pharmacology Unit, School of Medicine, Western Sydney University, Penrith, NSW, Australia; ^2^NICM Health Research Institute, Western Sydney University, Penrith, NSW, Australia; ^3^Department of Neurology, Shanghai Fourth People’s Hospital, Tongji University, Shanghai, China; ^4^School of Science and Health, Western Sydney University, Penrith, NSW, Australia; ^5^Behavioral Neuroscience Unit, School of Medicine, Western Sydney University, Penrith, NSW, Australia

**Keywords:** chronic microglia activation, motor function, degeneration, interleukin-6, cerebellum, microglia

## Abstract

Chronic microglial activation is a prominent feature of many chronic neurodegenerative diseases, including Parkinson’s and Alzheimer’s disease. To investigate the effects of chronic microglial activation on cerebellar structure and motor function throughout the lifespan, the transgenic GFAP-IL6 mouse model was used. The aim of the study was to examine inflammatory markers and neuronal degeneration while simultaneously characterizing the motor performance of GFAP-IL6 mice at 3, 6, 14, and 24 months of age in comparison to WT (C57BL/6) mice. In respect to markers of neuroinflammation in the cerebellum, increased numbers of Iba1^+^ microglia were observed as early as at 3 months of age. In addition, TNF-α levels proved to be significantly higher in the GFAP-IL6 compared to WT mice at all time points. A difference in cerebellar volume between the GFAP-IL6 and WT mice was observed later in life, starting at 6 months and increasing to a loss of about 50% in aged (24 months old) GFAP-IL6 mice. Synaptic deficits were also assessed by using pre- (synaptophysin) and post-synaptic (PSD95) markers. While synaptophysin levels remained unchanged, PSD95 levels decreased in the aging GFAP-IL6 mice compared to their WT littermates from 14 months onward. To assess the effect of microglia activation and neurodegeneration on behavior, a variety of motor function tests, semi-quantitative cerebellar ataxia score, accelerod, beam walking, and open field tests were performed. An age-dependent difference between the genotypes was observed in many of the motor function tests. For example, reduced performance on the accelerod and higher ataxia scores were observed at 6 months of age, followed by the beam walking test showing differences at 14 months of age. In summary, this study constitutes a comprehensive, age-dependent examination of inflammatory, synaptic and neurodegenerative changes in the brains of GFAP-IL6 mice leading to a deterioration in motor performance. The results also indicate that early chronic microglia activation in the GFAP-IL6 mouse leads to observable cerebellar volume loss and motor deficits later in life.

## Introduction

Normal aging of the brain is described by cortical and hippocampal volume loss, as well as increased demyelination, contributing to memory and learning deficits (Guha et al., 2016; Yoo et al., 2016; Xiao et al., 2017). However, chronic microglial activation (often referred to as “neuroinflammation”) accelerates age-related cognitive decline in many neurodegenerative diseases.

Microglia, the macrophages of the central nervous system (CNS), represent approximately 10% of all brain cells. Their main roles include providing nourishment and support to neurons, clearing debris, and being the first responders to foreign stimuli (Wake et al., 2013). Chronic microglial activation (T-cell independent neuroinflammation) has been described in many neurodegenerative diseases such as chronic traumatic encephalopathy, amyotrophic lateral sclerosis, Parkinson’s disease (PD), and Alzheimer’s disease (AD) (Mrak and Griffin, 2005; Eikelenboom et al., 2010; Kern et al., 2015).

Many neuroinflammatory markers are elevated in these diseases, as evidenced by up-regulation of oxidative stress markers, as well as pro-inflammatory chemokines and cytokines (Lee et al., 2000; Godbout et al., 2005).

During aging and other conditions associated with neuroinflammation, the morphology, activation state and number of microglia change. For example, activated microglia display an increased soma volume and retraction of processes (Sierra et al., 2007; Cerbai et al., 2012; Wong, 2013). Furthermore, microglial function has been shown to decline with age, impairing the cells ability to survey the brain and respond to insults (Hefendehl et al., 2014).

Aging, and possibly chronic neuroinflammation, are also associated with motor decline, contributing to functional limitation and disability in the elderly (Fried and Guralnik, 1997; Rantanen et al., 2012). The cumulative factors contributing to the loss of motor function during aging are not known, but low-grade chronic neuroinflammation has been investigated as one of the most potent contributors. Pro-inflammatory cytokines (such as IL-1, IL-6, and TNF) were shown to increase with age, and are associated with motor function loss (Dinarello, 2000; Franceschi, 2007; Franceschi et al., 2007). For instance, higher TNF-α levels were shown to be associated with decreased brain volume in older adults (Braskie et al., 2014).

It has been suggested that the progression of many neurodegenerative diseases, including AD, is driven by a cycle of self-perpetuating inflammatory neurotoxicity by following a mechanistic cascade (Block et al., 2007). Various inflammatory triggers can lead to the initial microglial activation. These triggers can be peripheral (for example, systemic infections or peripheral chronic inflammation) or central (for example degenerating/dying neurons or amyloid deposits). In the GFAP-IL6 mouse model, the trigger is the brain-specific production of the cytokine IL6 by astrocytes. Activated microglia release neurotoxic factors such as cytotoxic cytokines (like TNF-α and reactive oxygen/nitrogen species), which in turn cause damage to neighboring neurons. Damaged neurons then produce danger-associated molecular patterns, which activate microglia and perpetuate the cycle.

In the GFAP-IL6 transgenic mouse model of chronic neuroinflammation, IL6 is overexpressed in the brain, under the control of the GFAP promoter. The highest levels of IL6 expression have been observed in the cerebellum, approximately 50 times more than in their WT counterparts (Quintana et al., 2009). Homozygous GFAP-IL6 mice develop an aggressive pathology with tremor, ataxia, and seizures, and die prematurely. Heterozygous GFAP-IL6 mice, which were used in this study, however, are phenotypically milder, presenting with abnormalities such as astrocytosis, microgliosis, angiogenesis, and neurodegeneration (Campbell et al., 1993; Giralt et al., 2013).

Using this aging accelerated model of chronic neuroinflammation, we set out to investigate the effects of the pro-inflammatory cytokine IL-6 on microglial activation, cerebellar volume, neurodegeneration and motor function loss during the lifespan of the GFAP-IL6 mice.

## Materials and Methods

### Animals

Experiments were performed with heterozygous GFAP-IL6 and their wild-type (C57BL/6) littermates (both genders) aged 3, 6, 14, and 24 months (*n* = 9–20/age group/genotype, Table 1). Generation of the GFAP-IL6 transgenic mice was performed as described previously (Campbell et al., 1993). The genotype of all mice was verified by PCR analysis of DNA collected from ear notches as described previously (Campbell et al., 1993). Animals were housed in a temperature-controlled environment, in individually ventilated cages (IVC), with a normal 12:12 h light-dark cycle, with free access to food and water. Basic enrichments, domes, nesting material (crinkle nest) and a pipe tube (polyvinyl chloride) were provided. All procedures were approved by the Western Sydney University Animal Care and Ethics Committee (Animal Research Authority #A10057) and follow the “Guidelines to Promote the Wellbeing of Animals used for Scientific Purposes” as laid out by the National Health and Medical Research Council of Australia.

**Table 1:** Number of animals used for each behavioral experiment.

	3 months		6 months		14 months		24 months	
	C57BL/6	GFAP-IL6	C57BL/6	GFAP-IL6	C57BL/6	GFAP-IL6	C57BL/6	GFAP-IL6
Weight	13	13	11	12	10	14	11	10
Ataxia	13	13	11	12	10	14	11	10
Accelerod	20	20	16	18	21	23	20	18
Beam walking (time)	19	20	11	12	9	13	20	18
Beam walking (foots lips)	10	11	10	12	9	13	20	18
Open field	19	20	11	12	6	7	10	18

### TNF-α ELISA

Homogenization of the cerebellum from the mouse brain was performed using a lysis buffer [100 mM Tris (pH 7.4), 150 mM NaCl, 1 mM EDTA, 1 mM EGTA, 1% Triton-x 100 (v/v) and 0.5% sodium deoxycholate] with freshly added protease and phosphatase inhibitors (Sigmafast, Sigma) in a homogenizer. The lysate was centrifuged at 14,000 rpm for 20 min at 4°C and total protein estimation of the supernatant was tested using the Bradford reagent (Bio-Rad) at 595 nm (UV-absorption). The diluted brain samples of an equal amount of protein were used for determination of TNF-α using a sandwich ELISA according to the manufacturer’s instructions (Peprotech) with a small number of modifications. In brief, 100 μL of capture antibody (diluted in carbonate/bicarbonate buffer (pH 9.5) to 0.65 μg/mL) was incubated in a 96-well plate overnight at room temperature. After four washes [0.05% Tween-20 in PBS (pH 7.4)], 300 μL of blocking buffer (1% BSA in PBS) was added to each well and the plate was incubated for 1 h at room temperature. 100 μL of TNF-α standard (serially diluted from 0 to 10,000 pg/mL in diluent) or diluted brain homogenate was added to the wells in triplicate after four washes and incubated for 2 h at 37°C. The wells were washed and 100 μL of detection antibody (diluted to 0.2 μg/mL in diluent) was added to each well. After 1.5 h incubation at 37°C, the wells were washed and 100 μL of avidin-peroxidase conjugate (diluted to a ratio of 1:2,000 in diluent) was added to each well. The wells were incubated for 30 min before being washed. Finally, 100 μL of 3,3,5,5-tetramethylbenzidine (TMB) liquid substrate solution (3 mg TMB dissolved in 1 mL DMSO, supplemented with 9 mL phosphate-citrate buffer and 10 μL 30% H_2_O_2_) was added. Absorbance was measured at 655 nm in a POLAR star Omega microplate reader (BMG Labtech, Mornington, VIC, Australia) every 5 min. After 25 min, the reaction was stopped using 0.5 M sulfuric acid, and then the absorbance was measured at a wavelength of 455 nm. Absorbance readings were converted to the corresponding concentrations by using a standard curve constructed with defined concentrations of TNF-α. Samples were measured in triplicate, and all the values were used for the statistical analysis.

### Histology

#### Nissl Staining

Mice were anesthetized with isoflurane at the end of each time point and transcardial perfusion was performed. The heart was flushed with 30 mL of 0.9% normal saline using a peristaltic pump, followed by 60 mL of 4% paraformaldehyde (Merck) in 0.1 M phosphate buffer (PB). The brains were removed and post-fixed in 4% paraformaldehyde for at least 2 h at 4°C, and then transferred to 30% sucrose (in 0.1 M PB solution) for cryoprotection. After the brains sank to the bottom of the container, they were cut into 40 μm thick coronal sections using a Leica CM 1950 cryostat. The first series of sections were mounted onto 1% gelatin-coated glass slides. They were dehydrated in ethanol gradient (50%, 70%, 95%, 100%, 30 s each) before they were de-fatted in 95% ethanol with 5% acetic acid pre-warmed to 55–60°C for 15–20 min. Slides were then incubated in 0.2% cresyl violet solution pre-warmed to 55–60°C for 2–3 min. After being rinsed with 70% ethanol for 30 s and 95% ethanol for 1 min, sections were then differentiated in 95% ethanol with 0.5% acetic acid for 10–15 min until the optimal color was observed. Sections were then dehydrated in gradient ethanol (70%, 95%, 100%, 5 min each) and cleared in xylene (10 min) before they were coverslipped with the DPX (Merck, 100579) mounting medium.

#### Immunohistochemistry

The second and third series of sections were used for immunohistochemical staining to visualize neurons and microglia. They were treated in 1% hydrogen peroxide (in 0.1 M PBS, 30 min) to quench the endogenous peroxidase. After three rinses with 0.01 M PBS, they were incubated in 1% normal goat serum for 3 h to block the non-specific binding sites. Sections were then incubated in primary antibody solution (rabbit anti-Iba1, 019-19741 from Wako Chemical, 1:500; mouse anti-NeuN, MAB377 from Merck, 1:1000) overnight at room temperature. After three rinses with 0.01 M PBS, they were incubated in the biotinylated secondary antibody solution (goat anti-mouse IgG for NeuN, 1:200, #62-6540, Thermo Fisher; goat anti-rabbit IgG for Iba1, 1:200, #65-6140, Thermo Fisher) for 2 h at room temperature. Sections were then rinsed and incubated in HRP conjugated streptavidin (1:1,000, #189733, Merck) for 2 h. The peroxidase reaction was carried out using a developer solution containing 0.4 mg/mL DAB and 0.0006% hydrogen peroxide dissolved in 0.1 M TBS. For the negative control, the primary antibodies were omitted, and all other steps carried out as described above. After the staining procedures, sections were mounted onto gelatin-coated slides and dehydrated and coverslipped with DPX mounting medium.

#### Stereological Analysis

For volume estimation, the area of interest (the cerebellum) was traced on the Nissl-stained sections at 2.5× magnification and the cerebellar volume was estimated using the Cavalieri method (Brown et al., 2000). An estimation of the absolute neuronal and microglial cell number in the cerebellum was counted on the immunohistochemically stained series of sections using the Optical Fractionator probe (West et al., 1991; Bastide et al., 2016). The contour of the cerebellum was first drawn on every eighth section under 2.5× objective. The size of the counting grid was 850 × 850 μm for neuronal counting and 1,000 × 1,500 μm for microglia counting. The size of the counting frame was 20 × 20 μm for neuronal counting and 100 × 100 μm for C57BL/6 and 50 × 50 μm for GFAP-IL6 microglia counting. The guard zone was set up to be 1 μm at the top and the bottom of the sections. Microglia were plotted on the screen using a marker as the focus moved from the top to the bottom of the sections using the 63× oil objective. This has led to the Gunderson coefficient error to be less than 0.1 in all cases (*m* = 1).

### Western Blot

Tissue samples from the cerebellum of mice brains were homogenized (10% w/v) in ice-cold lysis buffer [100 mM Tris (pH 7.4), 150 mM NaCl, 1 mM EDTA, 1 mM EGTA, 1% Triton-x 100 (v/v) and 0.5% sodium deoxycholate] with freshly added protease (Thermo Fisher, Cat No: A32963) and phosphatase inhibitors (Sodium orthovanadate, sodium fluoride, staurosporine), using a motor-driven homogenizer (Thomas Scientific) on ice. The lysate was centrifuged at 14,000 rpm for 20 min at 4°C and total protein estimation of the supernatant was tested using the Bradford reagent (Bio-Rad) at 595 nm (UV-absorption). After protein quantification, tissue extracts (25 μg) were boiled in Laemmli sample buffer at 95°C for 10 min, followed by 10% SDS-PAGE electrophoresis at 90 V for 1.5 h, electro-transferred to nitrocellulose membranes for 2 h at 100 V. Non-specific protein binding was blocked with 5% milk in 0.1% Tween TBS. Membranes were incubated overnight at 4°C with a mouse monoclonal antibody to PSD95 (0.5 μg/mL, # MABN68, Merck) or a mouse polyclonal antibody to Synaptophysin (1:500, # ab8049, Abcam) in 0.1% Tween TBS. Membranes were then washed and incubated for 1 h with an HRP-conjugated secondary antibody (1:10,000) in 0.1% Tween TBS. Membranes were then washed and immunoreactivity was visualized with a chemiluminescence detection method (ECL, Merck Millipore) using Imagelab software (Bio-Rad). Optical densitometry was performed on each targeted band (Synaptophysin: 38 kDa; PSD-95: 95 kDa) using a computerized image analysis system ImageJ (NIH) software.

### Behavioral Experiments

#### Handling and Habituation

Handling was performed for three consecutive days for 2 min per mouse in the animal facility. After the daily handling, the mice were transported to the testing room for habituation. On testing days, animals were placed in the testing room for at least 30 min prior to testing in their home-cages. The same animals were assessed across all behavioral tests with 24–48 h of interest intervals, but separate animals were tested for each age group. Hence, at ages 3, 6, 14, and 24 months, four different groups of animals underwent the behavioral test battery. During the testing day, the order was chosen by taking into consideration gender and cage separation effect, hence males from the cohort were tested first, followed by females, and a maximum of three animals were tested from a cage (Table 2).

**Table 2:** Behavioral paradigm for motor function testing.

Day	Time (AM)	Behavioral paradigm
1	8:00–12:30	Handling I
2	8:00–12:30	Handling II
3	8:00–12:30	Handling III
4	8:00–12:30	Introduction to transport and experimental room
8	8:00–12:30	Open field
15	8:00–12:30	Walking beam training day 1
16	8:00–12:30	Walking beam training day 2
17	8:00–12:30	Walking beam training testing day
19	8:00–12:30	Accelerod
22	8:00–12:30	Physical and Composite Score

#### Physical Exam

The physical exam included general health observations corresponding to several parameters, such as the presence of whiskers, the appearance of fur, weight, piloerection, wounds, patches of fur on face, which was based on a semi-quantitative scale (0–4) (Crawley, 1999; Karl et al., 2003). These observational protocols were adapted from Erwin physical methodology (Irwin, 1968).

#### Semi-Quantitative Cerebellar Ataxia Scoring (SQCA)

The ataxia level was measured as the ability to perform the ledge test, expression of hind limb clasping, gait, and kyphosis. Every measurement was scored on a scale of 0–3 levels, with 0 representing the normal phenotype and 3 representing the most severe expression of the phenotype. Each test was performed three times to assess reproducibility (Guyenet et al., 2010). The SQCA contains the individual tests listed below (2.5.3.1–2.5.3.7).

##### Ledge test

The ledge test is a test for balance and coordination which is impaired in ataxias and other neurodegenerative diseases. The mouse was placed on the ledge of the cage and the experimenter scored the ability of the mouse to walk on the edge, using its tail as a counterbalance and balance to descend gracefully back to the cage or table using their paws. This resulted in a score of 0. A score of 1 was characterized by foot slips while walking along the ledge, maybe the tail presents rigidity and not able to counterbalance well but still able to walk and descend using their paws. A mouse with a score 2 was not effectively using its hind legs or landed on its head rather than its paws when descending the cage. When the mouse fell off the ledge, or nearly so, or when attempting to descend back to the cage, then a score of 3 was given (Guyenet et al., 2010).

##### Hindlimb clasping test

The hindlimb clasping test also follows a 3-level scoring system. Changes in this test have been observed in neurodegenerative disease and in some types of cerebral ataxia mouse models (Chou et al., 2008). The mouse was held by its tail’s base and suspended for 10 s in front of the experimenter. If the hindlimbs were splayed outward away from the abdomen consistently the score was 0. If one of the hindlimbs is retracted toward the abdomen for more than 50% of the observation time, this resulted in a score of 1. If both of the hindlimbs were partially retracted toward the abdomen for more than 50%, this resulted in a score of 2. If both hindlimbs were entirely retracted for more than 50% of the time, the mouse received a score of 3 (Thomas et al., 2006; Guyenet et al., 2010).

##### Kyphosis

The expression of this phenotype “kyphotic mouse” was typically seen as a curvature of the dorsal spine (hunchback) and it has been observed in several neurodegenerative diseases (Thomas et al., 2006). This phenotype is mostly caused by a lack of muscle strength. The experimenter positioned the mouse on a flat surface and observed the spine while the animal walked. A normal mouse (scored 0) walked with a straight spine. A score of 1 was given to an animal which showed a mild kyphosis but was still able to straighten its back. A scored of 2 was given if the mouse was unable to fully straighten its spine and maintained persistent mild kyphosis during locomotion. If the mouse maintained a constant pronounced kyphosis, it received a score of 3 (Thomas et al., 2006; Guyenet et al., 2010).

##### Gait

The objective of this test was to score coordination and muscle function of the walking pattern (gait). The mouse was placed on a flat surface and the experimenter observed the mouse walking. A score of 0 or normal was assigned to a mouse that walked normally, with no limping, its weight supported by all limbs and the abdomen did not touch the ground (3 mm normal distance). A score of 1 was characterized by the presence of a light, mild tremor or some limping. If the mouse showed a severe tremor or limping, lower pelvis, or feet pointing away from the body while walking, it received a score of 2. If the mouse was unable to walk forward and drags its abdomen along the ground, it received a score of 3 (Guyenet et al., 2010).

#### Accelerod

This test assesses the coordination and balance in rodents using an accelerating, rotating 9 cm diameter rod apparatus (Ugo Basile, Biological Research Apparatus, Varese, Italy), and uses “time to fall” as the readout. Each mouse was placed on the apparatus at a steady speed of 5 rpm. The speed was gradually increased from 5 rpm to 40 rpm over a period of 300 s, and the latency until the mouse fell off the rod was measured (with a maximum cut off time of 300 s). Each mouse was subjected to three trials a day with a 45 min inter-trial interval.

#### Beam Walking Test

This protocol is a sensitive measure of fine coordination, muscle function, and balance (Carter et al., 2001; Southwell et al., 2009; Luong et al., 2011). The objective of the assay was to measure the ability of the mice to cross the narrow walking beam. Performance on the beam was quantified by measuring the time it takes for the mice to traverse the beam and the number of foot slips that occurred while crossing. The beam apparatus was 1 m long and elevated by 52 cm, with an upper surface of 0.4 cm width and a lower surface of 1.5 cm width. The home cage and nesting material were located at the end of the beam in order to attract the mouse. During the first training session, the home cage was moved toward the mouse to direct them across the beam (first and second day). A lamp was located at the beginning of the beam (with a 70 watt light bulb, 800 lux) to encourage the mouse toward the safety of the darker home cage. The time to traverse the 81 cm distance from the starting point to the home cage was recorded by a blind observer. The timer was started when the nose of the mice passed the starting mark and stopped when the nose of the mice entered the home cage. Each day the mice crossed the beam three times. The average of the two best times on the testing day (third day) were plotted and analyzed. Inter-trial test interval was 30 s and the beam was thoroughly cleaned with 70% ethanol after each mouse was tested. The number of foot slips was measured using the video recording of the testing day, as the camera was located behind the starting point of the beam.

#### Open Field Test

The open field apparatus consisted of a square arena (44 cm × 44 cm) with transparent walls (30.5 cm height). Luminosity was set at 700 lux. The arena was divided into a peripheral area (9 cm wide from the edge of the wall), and a central area (26 cm × 26 cm). When the mice were placed in a corner of the arena, they preferentially spent the majority of their time in the periphery. The natural instincts of exploring the new environment but avoiding open spaces allowed the assessment of motor activity (e.g., total distance traveled) (Crawley, 1985). A monochrome camera (DMK72BUC02, The Imagining Source^®^) was connected to a computer for recording motor activity. Mice were automatically tracked and measured at a rate of 10 frames/s, using video-tracking software (ANY-maze^®^, Stoelting Co., United States). The total distance traveled was used as an indication of general locomotion activity, which was measured in the open field test for 10 min.

### Statistical Analysis

For all our behavioral analysis that passed the D’Agostino and Pearson normality test, parametric three-way ANOVA was first used with the variable factors ‘age,’ ‘genotype,’ and ‘gender.’ We did not detect any sex-effects (*p* > 0.05) and therefore combined male and female data sets for further analysis using two-way ANOVA for ‘genotype’ and ‘age’ with Tukey’s correction for multiple comparisons. In case of significant interactions between ‘genotype’ and ‘age,’ data was split by the corresponding factors. For data that did not pass the D’Agostino and Pearson test for normal distribution, we used the non-parametric test of Kruskal–Wallis, followed by Dunn’s multiple comparison test, which was performed with Prism GraphPad (version 8). This only applied to the ataxia score data set, where the ataxia score was the dependent variable.

**Table 3:** Summary of the absolute estimated microglia numbers in the cerebellum, presented with SD and SEM.

	3 months		6 months		14 months		24 months	
Genotype	C57/B6	GFAP-IL6	C57/B6	GFAP-IL6	C57/B6	GFAP-IL6	C57/B6	GFAP-IL6

*n*	6	6	4	5	6	6	5	5
Microglia number	172,776	1,586,909	271,067	1,610,356	230,279	1,168,631	197,551	525,682
SD	2,5597	153,604	53,388	189,736	51,940	138,750	53,188	101,651
SEM	10,450	62,709	26,694	84,853	21,204	62,051	23,786	45,460

Histological and molecular (Elisa and Western Blot) quantification analysis results were analyzed using two-way ANOVA with *F* test to determine whether the factors overall influenced the outcome significantly, and Tukey’s correction was used for multiple comparisons. Differences were regarded as statistically significant when *p* < 0.05. Three-way ANOVA analysis was performed using SPSS 22.0 for Windows. The two-way ANOVA with multiple comparisons was performed with Prism GraphPad (version 8). Statistical outliers were defined where the values were smaller or bigger than ±2.5 times of standard deviations from the overall mean (Miller, 1991). Results are presented as mean ± SEM.

## Results

### Comparison of Cerebellar TNF-α Levels Between WT and GFAP-IL6 Mice

**FIGURE 1:**
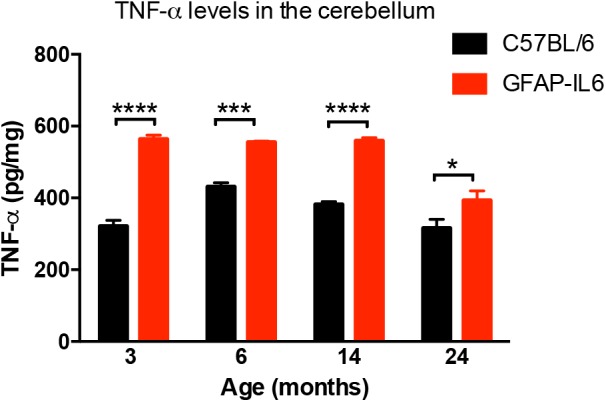
TNF-α levels measured in the cerebellum of WT (C57BL/6) and GFAP-IL6 animals. Data presented as mean ± SEM. TNF-alpha levels were significantly higher in all the age groups of the GFAP-IL6 mice when compared to their non-transgenic littermates. ^∗^ indicates *p* < 0.05, ^∗∗∗^ indicates *p* < 0.001, and ^∗∗∗∗^ indicates *p* < 0.0001; two-way ANOVA, with Tukey *post hoc* test.

In order to determine the levels of the pro-inflammatory marker TNF-α in both WT and the GFAP-IL6 groups, TNF-α was quantified using a commercial TNF-α ELISA. GFAP-IL6 mice showed a significantly higher level of TNF-α compared to their WT counterparts in all age groups. This difference was largest at 3 months (WT: 321 ± 16.5 pg/mg and GFAP-IL6: 563 ± 11.6 pg/mg; *p* < 0.0001), but remained significant throughout all age groups (at 6 months C57BL/6: 431 ± 11.2 pg/mg; GFAP-IL6: 555 ± 3.15 pg/mg; *p* = 0.0005, at 14 months C57BL/6 382 ± 7.71 pg/mg and GFAP-IL6: 559 ± 8.63 pg/mg; *p* < 0.0001 and 24 months C57BL/6: 316 ± 24.8 pg/mg and GFAP-IL6: 393 ± 26.4 pg/mg; *p* < 0.002, Figure 1). Detailed statistical analysis using a two-way ANOVA revealed that both age [*F*(3,118) = 25.96, *p* < 0.0001] and genotype [*F*(3,118) = 149.8, *p* < 0.0001] had significant effects on TNF-α, and additionally, a significant interaction between them was observed [*F*(3,118) = 7.887, *p* < 0.0001].

### Comparison of Cerebellar Iba1^+^ Microglia Numbers Between WT and GFAP-IL6 Mice

**FIGURE 2:**
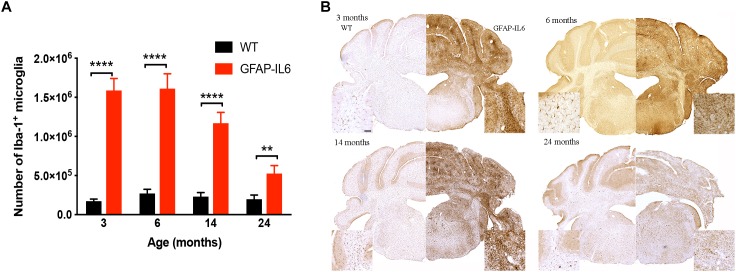
Quantitative analysis of microglial cell numbers in the cerebellum. **(A)** We found significant differences between wild-type and GFAP-IL6 animals at all time points in the absolute microglia numbers. **(B)** Images showed corresponding increased microglia numbers in the cerebellum. Data presented as mean ± SEM. ^∗∗^ indicates *p* < 0.01 and ^∗∗∗∗^ indicates *p* < 0.0001; two-way ANOVA, with Tukey *post hoc* test.

To determine a difference in the number of Iba1^+^ microglia between WT and GFAP-IL6 mice in the cerebellum, immunohistochemistry and stereological counting was conducted. In the GFAP-IL6 mice, the number of Iba1^+^ microglia in the cerebellum was determined to be 1,586,909 ± 153,604 in 3-month-old mice and 1,610,356 ± 189,736 in 6-month-old mice. In the older GFAP-IL6 mice, Iba1^+^ microglia numbers were reduced to 1,168,631 ± 138,750 at 14 months and further to 525,682 ± 101,651 at 24 months of age (Figure 2 and Supplementary Table S1). The number of microglia in the WT mice only changed with age from 172,776 ± 10,450 at 3 months of age, 271,067 ± 26,694 at 6 months of age, 230,279 ± 21,204 at 14 months of age and finally 197,551 ± 23,786 at 24 months of age. Further statistical analysis using two-way ANOVA demonstrated that both age [*F*(3,34) = 55.1, *p* < 0.0001] and genotype [*F*(1,34) = 850, *p* < 0.0001] had a significant effect on the number of Iba1^+^ microglia, with a significant interaction with each other [*F*(3,34) = 51.8, *p* < 0.0001] (Table 3).

When using Tukey’s corrected multiple comparisons, the number of Iba1^+^ microglial cells was significantly higher in the GFAP-IL6 groups at all time points compared to the wild-type controls [7.9 times in the 3-month group (*p* < 0.0001), 4.9 times in the 6-month group (*p* < 0.0001), 3.8 times in the 14-month group (*p* < 0.0001), and 1.7 times in the 24-month group (*p* = 0.001)]. The number of microglia was comparable within the age groups when assessing WT animals alone.

### Comparison of Cerebellar Volume Between WT and GFAP-IL6 Mice

**FIGURE 3:**
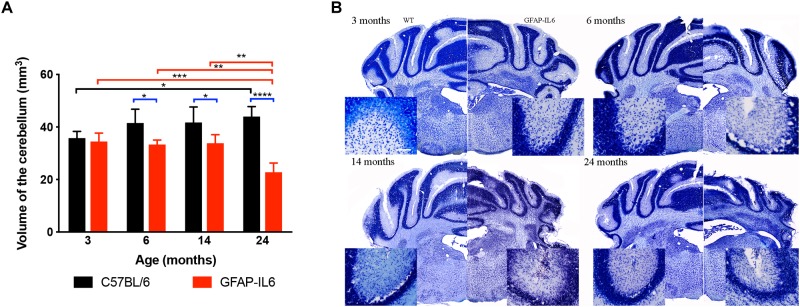
Decreased cerebellar volume at 6, 14 and 24 months. **(A)** The cerebellar volume of the GFAP-IL6 was found to be significantly decreased from 6 months compared to their WT littermates **(B)** Nissl stained representative sections at each time point demonstrating decreased cerebellar volumes from 6 months on in the GFAP-IL6 animals. Inserts showing the cellular disintegration of the cerebellar cell layers starting from 6 months of age. Data presented as mean ± SEM. ^∗^ indicates *p* < 0.05, ^∗∗^ indicates *p* < 0.01, ^∗∗∗^ indicates *p* < 0.001, and ^∗∗∗∗^ indicates *p* < 0.0001; two-way ANOVA, with Tukey *post hoc* test.

To investigate the extent of cerebellar damage in the GFAP-IL6 mice, the cerebellar volume was calculated using the Cavalier Estimator. Two-way ANOVA revealed a significant effect of genotype [*F*(1,35) = 62.3, *p* < 0.0001], but not of age [*F*(3,35) = 2.84, *p* = 0.052]. There was a significant interaction between the age and genotype effect [*F*(3,35) = 11.3, *p* < 0.0001]. Furthermore, we used multiple comparisons which showed that at the age of 3 months, GFAP-IL6 mice did not show any difference in cerebellar volume from their wild-type counterparts. However, GFAP-IL6 mice at the age of 6, 14, and 24 months showed a significantly decreased cerebellar volume compared to their wild-type counterparts, which was, in part, due to an increase in cerebellar volume of the WT mice (Figure 3A). At 6 months, the cerebellar volume of GFAP-IL6 mice (33 ± 1.7 mm^3^) decreased by 21.42% compared to WT mice (42 ± 5.2 μm^3^) (*p* < 0.05); at 14 months, the cerebellar volume of GFAP-IL6 mice (35 ± 4.9 mm^3^) decreased by 16.67% compared to WT mice (42 ± 5.9 mm^3^) (*p* < 0.05) and at 24 months, the cerebellar volume of GFAP-IL6 (23 ± 3.5 mm^3^) decreased by 47.72% compared to WT (44 ± 3.8 mm^3^) (*p* < 0.0001). When analyzing the effect of aging on the genotypes, we found a significant difference between 3 (36 ± 2.5) and 24 (44 ± 3.8) months (*p* = 0.04), with the volume increasing by approximately 11% within the wild WT group. Within the GFAP-IL6 group, however, the volume of the cerebellum of 24-month-old mice was significantly smaller than that of the three younger age groups (6 months *p* = 0.004 and 14 *p* = 0.002), suggesting progressive neurodegeneration from 14 months onward (Figure 3B).

### Comparison of Synaptic Markers Synaptophysin and PSD95 Between WT and GFAP-IL6 Mice

In order to investigate whether chronic microglial activation in the GFAP-IL6 mice led to changes in synaptic markers expression (and if this was age-dependent), the presynaptic marker synaptophysin and the postsynaptic marker PSD95 were quantified in cerebellum using Western Blots.

**FIGURE 4:**
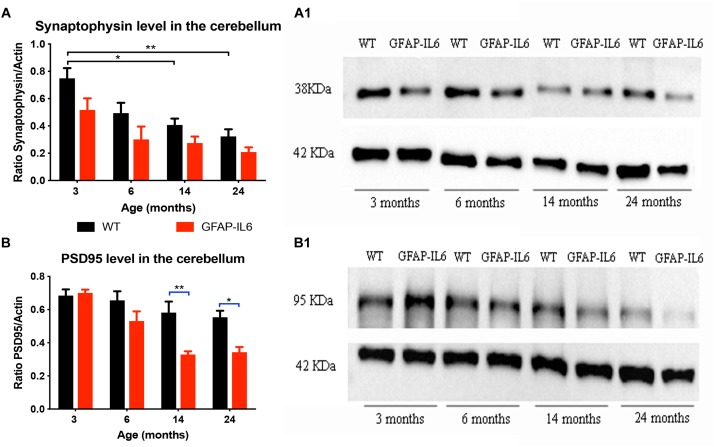
Increased IL6 overexpression decreased PSD95 levels in the cerebellum in the GFAP-IL6 during aging but did not significantly affected synaptophysin levels. Protein levels of PSD95 **(A)** and synaptophysin **(B)** were measured at 3, 6, 14, and 24 months of age in both WT and GFAP-IL6 animals (*n* = 5 for each groups) using Western Blot analysis. Panels **(A1,B1)** show an example blot from each group; for the summary data, each band was normalized to the respective α-actin band, and the normalized values are plotted relative to control levels. ^∗^*p* < 0.05, ^∗∗^*p* < 0.005; two-way ANOVA with Tukey *post hoc* correction.

For synaptophysin, levels declined in an age-dependent manner in both groups. Data indicated that both age [*F*(3,32) = 11.3, *p* < 0.001] and genotype [*F*(1,32) = 12.6, *p* = 0.001] had a positive effect on the synaptophysin protein levels, with no significant interactions [*F*(3,32) = 1.32, *p* = 0.81]. When using multiple comparisons (corrected by Tukey *post hoc* test), we did not find significant differences between WT and GFAP-IL6 at either of the time points. When examining the effect of aging on the genotypes, we found that the synaptophysin levels of the 3 months old WT group (0.75 ± 0.075) was significantly higher than the 14- (0.41 ± 0.04, *p* = 0.03) and 24- (0.32 ± 0.05, *p* = 0.002) -month-old group levels (Figure 4A,A1).

For PDS95, a significant difference in protein levels between WT and GFAP-IL6 mice was observed in the two oldest age groups. Detailed statistical analysis showed that both genotype [*F*(1,30) = 20.9, *p* < 0.001] and age [*F*(3,30) = 14.5, *p* < 0.001] had a significant effect on PSD95 levels with significant interaction [*F*(3,30) = 3.75, *p* = 0.02]. When using multiple comparisons (corrected by Tukey *post hoc* test), we found that the levels of PSD95 in the 14- (*p* = 0.005) and 24- (*p* = 0.05) -month-old animals were significantly reduced when compared to the WT (from 0.58 ± 0.06 to 0.33 ± 0.02 at 14 months and from 0.55 ± 0.04 to 0.34 ± 0.03 at 24 months). When we looked at the effect of aging on the PSD95 levels in GFAP-IL6 animals, we found that the 14- and 24-month-old mice significantly differed from the 3-month-old cohort (Figure 4B,B1).

To investigate whether the decrease in cerebellar volume in the GFAP-IL6 mice was caused by neuronal loss, NeuN staining was performed. Unfortunately, labeling of the NeuN positive neurons in the GFAP-IL6 animals became diffuse, showing a homogenous distribution of antigen localization within the cerebellum only. On the same sections, the NeuN staining showed optimal results in the brainstem areas, outside of the cerebellum. Hence, the recognition and separation of neurons was impossible using this staining method and antibody in the cerebellum. Therefore, we were unable to quantify the loss of neurons in the GFAP-IL6 groups, however, it was possible to quantify the number of neurons in the different age groups of the WT animals.

In the WT group, using one-way ANOVA, we found no significant differences in the number of neurons during aging [*F*(3,14) = 1.01, *p* = 0.4192], however, a trend of decreased neuronal numbers could be noticed. We found that the number of neurons stained by NeuN slightly decreased from 11,670,371 ± 2,043,358 at 3 months to 10,374,496 ± 779,635 at 24 months (Supplementary Figure S1).

### Determination of Changes in Motor Function in the GFAP-IL6 Compared to WT Mice

#### Comparison of Bodyweight Between WT and GFAP-IL6 Mice

**FIGURE 5:**
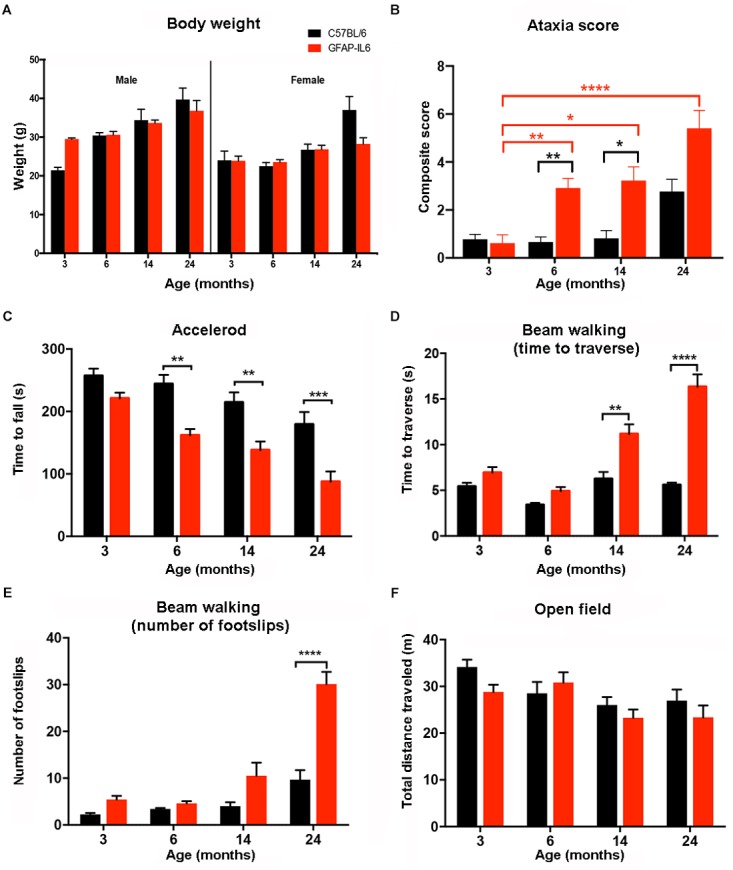
Summary of the behavioral analysis. **(A)** Body weight of male and female wild-type and GFAP-IL6 mice measured at 3, 6, 14, and 24 months of age. We found no significant differences in body weight in either male or female, wild-type and GFAP-IL6 animals at any time point. Three-way ANOVA, with Tukey *post hoc* comparison, was used. **(B)** Data shows the ataxia/composite score of WT (black columns) and GFAP-IL6 mice (red columns) with significant (*p* < 0.05) differences between genotypes at 6, and 14. Non-parametric Kruskal–Wallis test with Dunn’s multiple comparisons was used. **(C)** Accelerod performance of wild-type and GFAP-IL6 mice at various time points indicated significant differences at 6, 14, and 24 months between genotypes. Two-way ANOVA with Tukey *post hoc* correction was used. **(D)** Time to traverse on walking beam test results indicated significant differences between genotypes at 14 and 24 months and a significant decline of performance within the GFAP-IL6 group from 14 months on. Two-way ANOVA with Tukey *post hoc* correction was used. **(E)** Number of foot slips on the walking beam test results indicated significant differences within the GFAP-IL6 group affect by age at 24 months, WT animals did not perform significantly worse due to aging. Two-way ANOVA with Tukey *post hoc* correction was used. **(F)** Total distance traveled in the open field chamber revealed a general tendency of decline in mobility in all groups, with no significant differences though. Two-way ANOVA with Tukey *post hoc* correction was used. Data presented as mean ± SEM. ^∗^ indicates *p* < 0.05, ^∗∗^ indicates *p* < 0.01, ^∗∗∗^ indicates *p* < 0.001, and ^∗∗∗∗^ indicates *p* < 0.0001. Black columns represent C57BL/6 mice, and red columns represent GFAP-IL6 mice on all the data sets.

As increased body weight might contribute to the difference in motor function (especially balance tests), mice were weighed at the time of the motor function testing. Analysis of the data showed no significant differences for age [*F*(3,78) = 1.093, *p* = 0.357], genotype [*F*(1,78) = 2.562, *p* = 0.114] and gender [*F*(1,78) = 0.021, *p* = 0.886]. As expected, the females had a lower body weight in general than males in every age group. We found a tendency for the GFAP-IL6 males to have slightly lower body weight at 14 (34 ± 2.8 g) and 24 (37 ± 2.7 g) months compared to their non-transgenic littermates (35 ± 0.27 g and 40 ± 2.9 g). While the GFAP-IL6 females at 24 months (31 ± 3.4 g) had lower body weight than their WT counterparts (37 ± 3.5 g), these differences were not found to be significant, making it unlikely that difference in body weight between the genotypes could account for the difference in motor function (Figure 5A).

#### Comparison of Gross Locomotion Between WT and GFAP-IL6 Mice in the Open Field Test

To analyze gross locomotion of mice of both genotypes and different ages, the total distance traveled in the open field test chamber was measured. Older WT and GFAP-IL6 traveled shorter distances than younger mice [*F*(3,100) = 4.82, *p* = 0.0036]. Assessment of mobility of the mice in the open field suggested there was no interaction between genotype and age [*F*(3,100) = 1.042, *p* = 0.3773]. Genotype alone also did not contribute significantly to the differences in total distance traveled [*F*(1,100) = 1.839, *p* = 0.1781] (Figure 5F).

In summary, bodyweight and gross locomotion were not significantly different between WT and GFAP-IL6 mice, suggesting that differences in the following motor performance test were mostly due to neurodegenerative changes. A summary of all motor test results can be found in Supplementary Table S2.

#### Semi-Quantitative Determination of the Ataxia Score in WT and GFAP-IL6 Mice

Semi-quantitative ataxia scoring (measuring the ability to perform the ledge test, hind limb clasping, gait, and kyphosis) was performed in order to assess the severity of pathology of GFAP-IL6 mice across the lifespan (min of *n* = 10 per individual group). Every test was scored on a scale of 0–3 level, with 0 representing the normal phenotype and 3 representing the most severe expression of the phenotype. Using a non-parametric analysis (Kruskal–Wallis with Dunn’s multiple comparisons), we found significant differences between groups (*p* ≤ 0.0001).

When assessing the effect of aging on the WT animals, we found no significant difference within the WT groups at any of the time points. When assessing the effect of aging on the GFAP-IL6 mice, the ataxia score of the GFAP-IL6 cohort was not significantly different from the WT cohort at 3 months. However, ataxia worsened in older GFAP-IL6 mice, as the 6- (*p* = 0.007), 14- (*p* = 0.01), and 24- (*p* < 0.0001) -month-old GFAP-IL6 mice displayed significantly worse ataxia scores than the 3-month-old mice (Figure 5B). When genotype differences between the WT and GFAP-IL6 mice were analyzed, the ataxia score of the 6- (2.9 ± 0.4) and 14- (2.4 ± 0.54) -month-old GFAP-IL6 animals proved to be significantly worse than their WT littermates (0.9 ± 0.34 for 6 months and 1.2 ± 0.46 for 14 months, respectively) (Figure 5B).

#### Determination of Endurance on the Accelerod in WT and GFAP-IL6 Mice

To determine if WT and GFAP-IL6 mice develop deteriorating motor performance when they age, all groups were tested on the accelerod apparatus. In general, results show an age effect in both WT and GFAP-IL6 mice, with worsened performance in each group over time.

Analyzing our results using a two-way ANOVA test, we concluded there was a significant decline in the performance on the accelerod due to genotype [*F*(1,146) = 48.48, *p* < 0.0001] and age [*F*(3,146) = 18.82, *p* < 0.0001]. However, we found no significant interaction between genotype and age [*F*(3,146) = 1.45, *p* = 0.23]. Using Bonferroni-corrected multiple comparisons, a significant genotype effect was found at the 6- (*p* = 0.0068), 14- (*p* = 0.025), and 24-month (*p* = 0.00006) time points, with the GFAP-IL6 mice falling earlier from the apparatus (179 ± 17 s; 157 ± 17 s; 90 ± 16 s at 6, 14, 24 months, respectively) compared to WT mice (243 ± 18 s; 234 ± 28 s; 179 ± 20 s at 6, 14, and 24 months, respectively) (Figure 5C).

#### Determination of Balance and Coordination in the Beam Walking Test in WT and GFAP-IL6 Mice

The beam walking test determines balance and coordination skills in mice, assessed by two measures, including the time to traverse and the number of foot slips. Results indicated a significant interaction between age and genotype for both time to traverse [*F*(3,94) = 18, *p* < 0.0001] and the number of foot slips [*F*(3,79) = 11.7, *p* < 0.0001].

Genotype and age alone also significantly affected the results of both the time to cross [*F*(1,94) = 68.3, *p* < 0.0001 and *F*(3,94) = 29.8, *p* < 0.0001, respectively] and the number of foot slips [*F*(1,79) = 28.9, *p* < 0.0001 and *F*(3,79) = 35.6, *p* < 0.0001, respectively].

When the traversing time was analyzed using Bonferroni-corrected multiple comparisons to investigate the differences between genotypes and age groups, it increased significantly from 6 months of age in the GFAP-IL6 group (4.9 ± 0.46 at 6, 11 ± 1.1 at 14 and 16 ± 1.5 s at 24 months), while the wild-type animals crossed the beam in a comparable time limit (between 3.7 ± 0.33 and 5.6 ± 0.26 s). We found that at 14 and 24 months, the GFAP-IL6 mice took significantly longer to cross the beam compared to their WT counterparts (*p* < 0.01 and *p* < 0.0001) (Figure 5D).

Analysis of the number of foot slips on the walking beam also showed a progressive decline in this parameter in the aging the GFAP-IL6 mice. The number of foot slips increased from 4.6 ± 0.5 at 6 months to 11 ± 2.7 at 14 months until they reached 29 ± 2.3 at 24 months (*p* < 0.0001). The number of foot slips did not significantly change during the aging process of WT animals. We found significant differences between the wild-type (9.7 ± 2) and GFAP-IL6 (29 ± 2.3) group at 24 months of age (*p* < 0.001) (Figure 5E).

## Discussion

We are proposing the hypothesis that chronic microglial activation, driven by astrocyte-derived IL6 expression in the GFAP-IL6 mouse, initially leads to microglial proliferation and up-regulation of a variety of other pro-inflammatory markers, followed by a progressive decline in motor skills in later life. This study investigated the fine and gross motor function in GFAP-IL6 mice from 3 to 24 months of age and correlated these findings with the neuroinflammatory markers Iba1, the pro-inflammatory cytokine TNF-α and neurodegenerative changes of the cerebellum.

We carried out a stereological investigation of microglial numbers and tissue volume measurements in the cerebellum across the entire life span of the mouse. Using unbiased stereology, we found that from as early as 3 months of age, GFAP-IL6 mice exhibit increased numbers of Iba1^+^ microglia compared to wild-type mice, with this difference observable up to 24 months. The increase in microglia peaked at 3 months (9.18 times compared to wild-type), and gradually reduced to 5.9 times at 6 months, 5.07 times at 14 months, and finally declined to 2.66 times in the 24 months group. In addition, a GFAP-IL6 genotype-specific decrease in cerebellar volume was observed from 6 months of age.

Our results show a similar trend to a previous study, in which IL6 expression is detectable at 1 week of age, increases to maximal levels by 3 months, before declining at 8 months (Chiang et al., 1994). This indicates that the pro-inflammatory cytokine IL6 is initially able to activate microglia upon exposure and stimulate microglial proliferation and activation, as evidenced by densely stained cell bodies and fewer, shorter processes (Ayoub and Salm, 2003). However, during aging, the number of microglia (both activated and resting, labeled by Iba1) seems to decline, most likely together with general neurodegeneration and a loss of cerebellar tissue, as reported here. Hence, microglial activation, measured by upregulated microglial numbers, may have a reciprocal relationship to neuronal damage and behavioral performance.

We found the microglial number in the wild-type (C57BL/6) animals comparable to previous reports. Our results indicated that 3- and 6-month-old mice showed a similar density of microglia (4,914/mm^3^ and 6,585/mm^3^, respectively) to what has been reported previously (5,579 ± 793/mm^3^ microglia in the cerebellum of adult mice) (Vela et al., 1995). In another study, although not calculated, it can be estimated based on the density of microglia (8158 ± 1584 cells/mm^3^) and the measured volume of the cerebellum (24.5 ± 4.5 mm^3^) (Journiac et al., 2005), that the total number is approximately 199,871 microglia in the cerebellum. This is consistent with our stereological findings at 3 months and indicates the reliability of our microglial quantification methods.

This study also showed for the first time that overexpression of IL6 led to decreased cerebellar volume and possible neuronal loss in the cerebellum at the age of 24 months. This is consistent with previous studies which demonstrate that increased inflammation leads to neuronal loss (Tan et al., 1999; Brown and Neher, 2010). Though previous studies on GFAP-IL6 mice did not measure changes in neuronal number, we did observe pathological changes in the cerebellum as shown by collapsed and vacuolized dendrites, a condensed molecular layer of the cerebellum, and reactive gliosis. Others observed neuronal loss in the hippocampus of GFAP-IL6 mice at the age of 12 months (Campbell et al., 1993). These findings suggest that overexpression of IL6 leads to chronic neuroinflammation which in turn damages neurons, however, it has been suggested that this cell death is not mediated by direct toxicity of IL-6; rather, a downstream inflammatory process induced by IL-6 overexpression (Heyser et al., 1997). Based on our findings that cerebellar volume in GFAP-IL6 mice decreased after 6 months, while microglial numbers peaked at 3 months, we speculate that IL6 overexpression might incur neuronal damage through both direct and indirect pathways. As reported by Chiang et al. (1994), cytokines such as interleukin 1 alpha/beta, TNF-α are upregulated by overexpression of IL6 in the mouse brain. This is in accordance with our TNF-α measurements, indicating increased levels of the pro-inflammatory cytokine in the GFAP-IL6 animals at all time points. We found a significant decline in TNF-α levels in the GFAP-IL6 animals at the 14 and 24 months time points. This corresponds with declines in both absolute microglia numbers and indicating an overall tissue loss. We provided further indirect supporting data toward neuronal loss and degeneration in the GFAP-IL6 mouse, by measuring pre- and post-synaptic protein levels in the cerebellum. While PSD95 levels were decreased significantly in the GFAP-IL6 animals at 14 and 24 months of age, pre-synaptic (synaptophysin) protein levels were not significantly affected by genotype. With age, however, we found pre-synaptic protein levels to decrease significantly in both GFAP-IL6 and wild-type mice. As previously suggested (Han et al., 2017; Karoglu et al., 2017), our results indicate that both aging and pro-inflammatory cytokines alter synaptic protein dynamics, a finding that is modeled within the GFAP-IL6 brain.

Motor function and structural measures revealed a consistent pattern during the aging of the GFAP-IL6 mice. The composite score, accelerod performance and walking beam foot slip count all revealed a significant loss of motor function and structure in the GFAP-IL6 mice compared to WT mice, but only after 14 months of age. Our results are in agreement with those of Guyenet et al. (2010), who reported the composite score of non-transgenic mice from 3 to 10 months of age to be around 1. Previous reports on the accelerod, using 6- and 14-month-old GFAP-IL6 mice, showed a tendency toward reduced motor performance, but these results did not prove to be significant (Giralt et al., 2002). In this study, we demonstrated progressive, age-dependent impairments in accelerod motor performance in the GFAP-IL6 mouse compared with wild-type mice. The beam walking test was utilized to study fine coordination skills. When measuring time to cross and foot slip count, GFAP-IL6 mice were not significantly different from wild-type mice until 14 and 24 months. Furthermore, GFAP-IL6 animals performed significantly worse in the beam walking from 6 months onward if compared between time points, while the wild-type animals did not show a significant decline during aging. Previous studies showed that age effects motor coordination in mice (Allen and Cavanaugh, 2014), with older C57BL/6 mice (24 months) taking approximately the same time to cross the beam than middle-aged or young mice, however, with significantly more errors. While this study used a slightly modified, more challenging walking beam, our results regarding the crossing time of the aging C57BL/6 mice were in accordance with previous studies. Our study is the first to report that 14- and 24-month-old GFAP-IL6 mice spent significantly (two to three times) more time crossing the beam than their wild-type counterpart controls. The frequency of mistakes (number of foot slips) on the walking beam was also a sensitive measure in distinguishing genotypes at 24 months. Our study is the first to show age-dependent motor coordination decline due to increased chronic neuroinflammation. Together, the accelerod and walking beam findings from this study indicate a genotype-specific, age-dependent decrease in motor function and coordination in GFAP-IL6 mice, with substantial phenotypic effects at 24 months of age.

The open field test is widely used to measure the locomotor activity of mice. Our results indicated no significant differences in the overall distance traveled by either genotype or any age cohort. This is contrary to the findings of Shoji et al. (2016), who found significant differences between the younger and older mice within the first 5 min of the test, although their oldest animals were 12 months old. They noted no differences in total distance traveled during a 120 min experiment. Based on our findings we can conclude that the general locomotor activity of the GFAP-IL6 animals measured by the total distance traveled in the open field box was not affected.

As a limitation of the study, we must recognize that chronic microglial activation in the GFAP-IL6 mice is present from early ages (maybe even during the development of the fetus). Therefore, it is hard to describe the exact time course of microglial activation and whether these changes begin *in utero*. For this purpose, we would need to utilize an animal model with an inducible IL6 promoter, or the injection of an inflammatory stimulus (like LPS) at certain time points. Alternatively, GFAP-IL6 mice could be fed with a potent placenta and CNS permeable anti-inflammatory drug during gestation to avoid early neuroinflammation in offspring; withdrawing the drug at a later stage in order to allow the IL6 induced inflammatory process at a defined time.

In this model, the following questions were answered: Does microglial activation lead to tissue volume loss and presumably neurodegeneration (in the cerebellum) later in life? And: Does chronic microglial activation (particular in the cerebellum) lead to behavioral changes, such as a decline in motor function, later in life?

In summary, the increased microglia numbers and morphological changes observed from 3 months of age in the GFAP-IL6 mice do not seem to induce deterioration in coordination immediately, or at least not in the employed measures. However, during aging, at 6, 14, and 24 months, GFAP-IL6 mice have significantly worsened fine and gross motor coordination, measured by the accelerod and walking beam performance. While it is important to consider that neuroinflammation involves various pro-inflammatory cytokines, the IL-6 cytokine-induced neurological disease described in the GFAP-IL6 transgenic mouse suggests its suitability in closely replicating the neuroinflammatory and neurodegenerative processes underlying many neurological diseases and disorders.

These findings further demonstrate that neuroinflammation precedes neurodegeneration and functional loss of the motor circuit, and have substantial clinical relevance, providing a window of opportunity for early treatment of neurodegenerative diseases that are accompanied by neuroinflammation. The GFAP-IL6 represents a relevant phenotype to further our understanding of the effects of chronic neuroinflammation and offers a valuable opportunity to investigate potential anti-inflammatory and neuroprotective compounds *in viv*o.

## Data Availability

All datasets generated for this study are included in the manuscript and/or the Supplementary Files.

## Author Contributions

EG, HL, GN, TK, and GM contributed to conception and design of the study. FU, HL, GN, RA, MV, and DG contributed to data creation, collection and analysis. GN and AR organized the database. EG performed the statistical analysis. AR and EG wrote the first draft of the manuscript. AR, EG, DG, MV, HL, and GM wrote sections of the manuscript. All authors contributed to manuscript revision, read and approved the submitted version.

## Conflict of Interest Statement

The authors declare that the research was conducted in the absence of any commercial or financial relationships that could be construed as a potential conflict of interest.

## Supplementary Material

FIGURE S1 Quantitative and qualitative description of the NeuN labeled cells in the cerebellum. **(A)** The graph shows no significant differences within the wild-type animals at different time points in the number of neurons. Aging did not affect the cell numbers of the cerebellum in the WT mice, however the cellular disintegration of the GFAP-IL6 mice is evident from early on. **(B)** Representative images of brain sections stained with NeuN in both WT (left brain hemisphere) and GFAP-IL6 (right brain hemisphere) mice at 4 different time points. Data presented as mean ± SEM; two-way ANOVA, with Tukey *post hoc test*.

TABLE S1 Summary of stereological parameters used for Iba-1 positive microglia estimation in the cerebellum.

TABLE S2 Summary of ataxia score, accelerod, beam walking (time to cross and number of footslips) and total distance travelled in the open field test in C57BL/6 and GFAP-IL6 mice at 3, 6, 14, and 24 months of age represented as mean ± SEM.
