# Supplementary figures and images for: Chronic Microglial Activation in the GFAP-IL6 Mouse Contributes to Age-Dependent Cerebellar Volume Loss and Impairment in Motor Function
Source: Front Neurosci. 2019 Apr 3;13:303. doi: 10.3389/fnins.2019.00303 (PMC6456818; doi:10.3389/fnins.2019.00303)

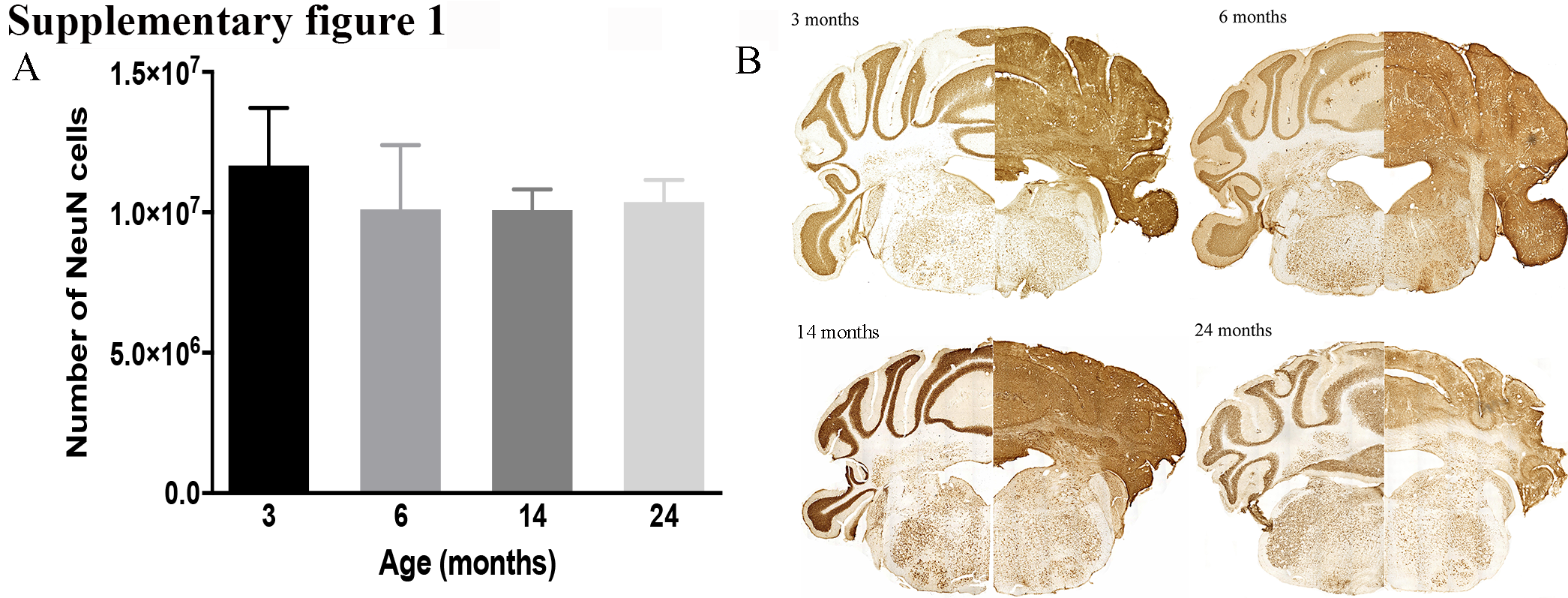

Supplement: FIGURE S1 — Quantitative and qualitative description of the NeuN labeled cells in the cerebellum. (A) The graph shows no significant differences within the wild-type animals at different time points in the number of neurons. Aging did not affect the cell numbers of the cerebellum in the WT mice, however the cellular disintegration of the GFAP-IL6 mice is evident from early on. (B) Representative images of brain sections stained with NeuN in both WT (left brain hemisphere) and GFAP-IL6 (right brain hemisphere) mice at 4 different time points. Data presented as mean ± SEM; two-way ANOVA, with Tukey post hoc test. [file Image_1.tif]
