# Supplementary material for: Chronic Microglial Activation in the GFAP-IL6 Mouse Contributes to Age-Dependent Cerebellar Volume Loss and Impairment in Motor Function
Source: Front Neurosci. 2019 Apr 3;13:303. doi: 10.3389/fnins.2019.00303 (PMC6456818; doi:10.3389/fnins.2019.00303)
Supplement: TABLE S1 — Summary of stereological parameters used for Iba-1 positive microglia estimation in the cerebellum. [file Table_1.DOCX]

Supplementary table 1. Summary of stereological parameters used for Iba-1 positive microglia estimation in the cerebellum.
