# Supplementary material for: Chronic Microglial Activation in the GFAP-IL6 Mouse Contributes to Age-Dependent Cerebellar Volume Loss and Impairment in Motor Function
Source: Front Neurosci. 2019 Apr 3;13:303. doi: 10.3389/fnins.2019.00303 (PMC6456818; doi:10.3389/fnins.2019.00303)
Supplement: TABLE S2 — Summary of ataxia score, accelerod, beam walking (time to cross and number of footslips) and total distance travelled in the open field test in C57BL/6 and GFAP-IL6 mice at 3, 6, 14, and 24 months of age represented as mean ± SEM. [file Table_2.DOCX]

|  | **3 months** | | **6 months** | | **14 months** | | **24 months** | |
| --- | --- | --- | --- | --- | --- | --- | --- | --- |
|  | ***C57BL/6*** | ***GFAP-IL6*** | ***C57BL/6*** | ***GFAP-IL6*** | ***C57BL/6*** | ***GFAP-IL6*** | ***C57BL/6*** | ***GFAP-IL6*** |
| **Ataxia score** | 0.78±0.21 | 0.62±0.35 | 0.94±0.34 | 2.9±0.40 | 1.2±0.46 | 2.4±0.54 | 3.0±0.52 | 4.9±0.84 |
| **Accelerod [s]** | 255±12 | 221±9.2 | 243±18 | 179±17 | 234±28 | 157±17 | 179±20 | 90±16 |
| **Walking beam (time to cross) [s]** | 5.4±0.40 | 6.9±0.61 | 3.7±0.33 | 4.9±0.46 | 5.2±0.67 | 11±1.1 | 5.6±0.26 | 16±1.4 |
| **Walking beam (number of footslips)** | 2.3±0.31 | 5.4±0.78 | 3.4±0.22 | 4.6±0.50 | 4.0±0.87 | 11±2.7 | 9.7±2.0 | 29±2.3 |
| **Open field (total distance traveled) [m]** | 34±1.6 | 29±1.6 | 29±2.5 | 31±2.2 | 26±1.7 | 23±1.8 | 27±2.4 | 23±2.5 |

Supplementary table 2. Summary of ataxia score, accelerod, beam walking (time to cross and number of footslips) and total distance travelled in the open field test in C57BL/6 and GFAP-IL6 mice at 3, 6, 14 and 24 months of age represented as mean ± SEM.
